# Female with Abdominal Wall Mass

**DOI:** 10.5811/cpcem.2017.6.34361

**Published:** 2017-10-03

**Authors:** Alex Koo, Scott Young

**Affiliations:** Madigan Army Medical Center, Department of Emergency Medicine, Joint Base Lewis-McChord, Washington

## CASE PRESENTATION

**Image 1:**
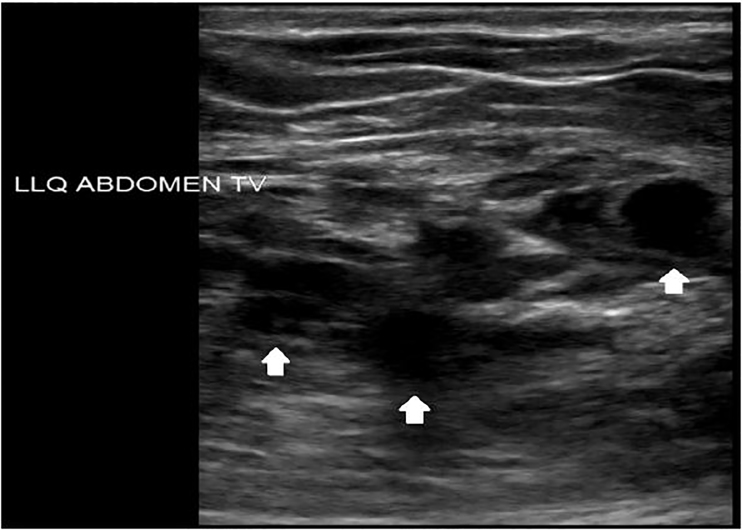
Transabdominal ultrasound of the mass in axial plane demonstrating anechoic, well-circumscribed structures (arrows).

**Image 2:**
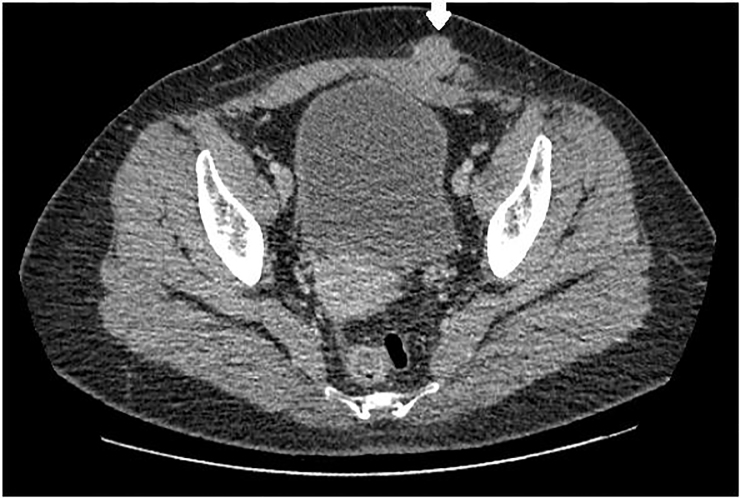
Computed tomography abdomen of the mass in axial plane demonstrating enhancing soft-tissue mass with irregular margins (arrow).

**Image 3:**
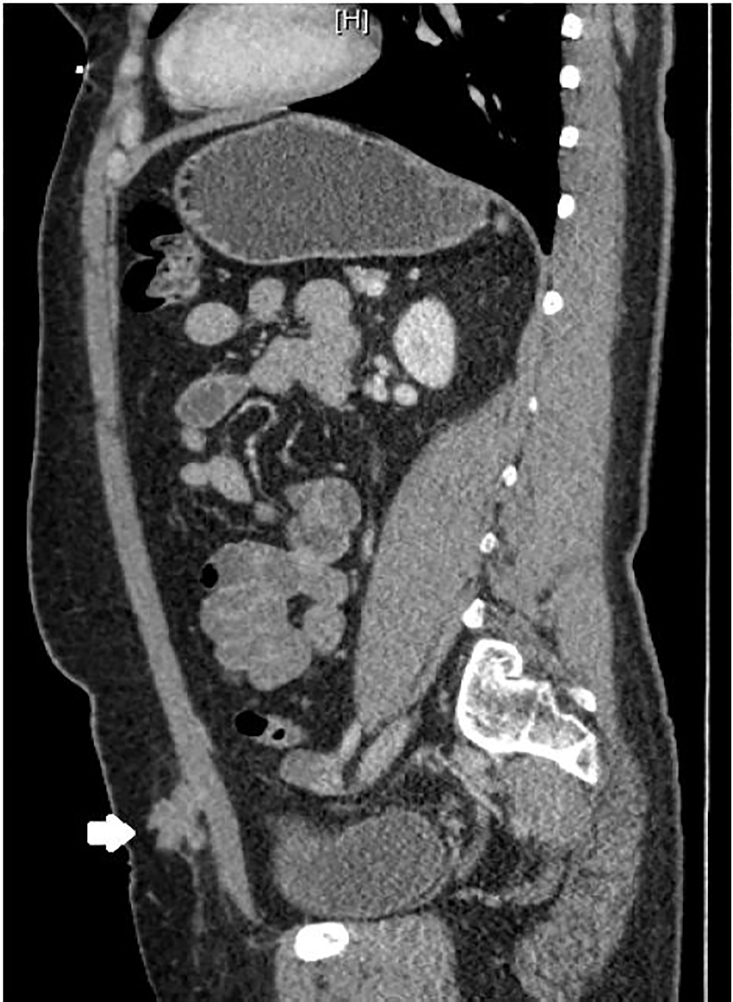
Computed tomography abdomen of the mass in sagittal plane demonstrating enhancing soft-tissue mass with irregular margins (arrow).

A 32-year-old female with a past surgical history of a low transverse Cesarean section presented to the emergency department with a left lower quadrant abdominal mass and pain. The patient had been seen previously by gynecology for similar pain, diagnosed with adhesions and undergone adhesion lysis without resolution. The pain occurred monthly coinciding with her menstrual cycle. On examination a palpable, mobile, and tender mass without erythema or fluctuance was noted in her superficial abdominal wall. Point-of-care ultrasonography with a 13–6 Megahertz linear transducer demonstrated a heterogeneous polycystic structure (Image 1). After discussion with the patient and radiology, computed tomography (CT) was performed (Images 2 and 3).

## DISCUSSION

### Abdominal Wall Endometrioma

CT imaging revealed a 2.4 × 2.0 × 2.8 cm enhancing abdominal wall mass with the same radiodensity as ovarian tissue. The patient was referred to general surgery for biopsy and excision.

Abdominal wall endometriomas (AWE) are ectopic endometrial tissue found superficial to the peritoneum, often developing adjacent to previous surgical scars.^1^ The majority of AWE have been reported after gynecological procedures, with an incidence of 0.08% after Cesarean sections.^1^,^2^ Cyclic pain with menses is present in less than half of the cases but can help predict the disease. AWE are often misdiagnosed as adhesions, hernias, abscess, lipomas, cysts, or tumors.^2^,^3^ Ultrasound may show cystic or polycystic structures, with CT demonstrating circumscribed enhancing masses and possible hemorrhage. AWE are less responsive to hormonal therapy than intrapelvic endometriomas, but are cured 95% of the time with surgical excision.^1^

**CPC-EM Capsule**

What do we already know about this clinical entity?

Abdominal wall endometriomas are rare but known complications adjacent to gynecological or obstetric abdominal incisions. Cyclical pain coinciding with menstrual cycles is common.

What is the major impact of the image(s)?

Abdominal wall endometriomas may be imaged by point-of-care ultrasound as heterogenous, cystic structures and, on computed tomography, will have similar densities to the ovaries.

How might this improve emergency medicine practice?

Abdominal wall endometrioma should be considered in the differential for abdominal wall masses with cyclical pain related to menstrual cycles.
